# Beyond polarization towards dynamic balance: harmony as the core of mental health

**DOI:** 10.3389/fpsyg.2023.1177657

**Published:** 2023-09-13

**Authors:** Antonella Delle Fave, Marié Philipina Wissing, Ingrid Brdar

**Affiliations:** ^1^Department of Pathophysiology and Transplantation, University of Milan, Milan, Italy; ^2^Africa Unit for Transdisciplinary Health Research (AUTHeR), North-West University, Potchefstroom, North West, South Africa; ^3^Department of Psychology, Faculty of Humanities and Social Sciences, University of Rijeka, Rijeka, Croatia

**Keywords:** mental health, well-being, maximization, harmony, balance

## Abstract

Worldviews are culturally derived assumptions that influence individual and collective behaviors, values, and representations of reality. The study of mental functions is not exempt from this influence, as reflected in scientific theories, methodological approaches, and empirical studies. Despite acknowledging the interplay of mental processes with developmental, environmental, and cultural dimensions, psychological research is still primarily based on quantitative methods, and on the conceptualization of mental phenomena as unfolding along polarized continua. A lively epistemological debate surrounds this approach, especially underscoring the risk of blurring the distinction between constructs derived from statistical models and real-life processes and experiences. Based on this debate and on recent empirical evidence derived from the positive psychology literature, this paper is aimed at proposing an integrated view of mental health, as a holistically patterned, contextually imbedded, and dynamic phenomenon changing over time and across life events, with harmony, harmonization and dynamic balance as core qualities. The heuristic potential of investigating the qualitative configuration patterns of mental health dimensions across individuals and groups, beyond their position along a quantitative continuum, is outlined. The development of more integrated approaches and methodologies to investigate mental health as a harmonization process, taking into account personal, contextual and developmental features, would be aligned with evidence derived from the integration of traditional nomothetic and ideographic approaches, and other life sciences. However, the development of a transdisciplinary line of research requires further inputs from different epistemological views, as well as higher attention to the potential contribution of different philosophical traditions.

## Introduction

In this paper we propose an integrated view of mental health, moving from its current conceptualizations and measurement as a set of positive dimensions (Keyes, 2003; Diener, 2006). We specifically discuss the limitations of the polarization approach characterizing this line of research, based on the assumption that positive mental health facets unfold along a continuum, with values ranging from a minimum to a maximum. Relying on multidisciplinary evidence derived from the study of real-life dynamics, we build a case for a new understanding of mental health as a holistically patterned and contextually imbedded phenomenon, changing over time and across life events, with harmony, harmonization and dynamic balance as core qualities. New epistemologies, transdisciplinary approaches and multi-method evaluation strategies are however required to advance knowledge in Psychology, and specifically in Positive Psychology, along this line of research.

In the next sections we will elaborate on the philosophical, theoretical and empirical underpinnings of the proposed new perspective.

## Mental health definitions and measurement

This section is focused on the construct of mental health, as defined and measured in the positive psychology literature. Based on culturally driven assumptions concerning the development and measurement of psychological constructs, mental health is currently defined as a quantifiable cumulative condition, that includes multiple elements unfolding on a bipolar continuum, ranging from minimum to maximum. Empirical findings however suggest that this polarized approach does not adequately reflect real-life experiences. We thus propose to define and measure mental health as an integrated set of multiple components, ceaselessly changing in intensity, interacting with each other, and contextualized in a given biocultural environment.

### Assumptions about psychological constructs and their polarity

The influence of culturally shaped worldviews on individual and collective behaviors, values and representations of reality is a core tenet in philosophy. Psychologists are not exempt from this influence. Being engaged in the exploration of phenomena that are not directly observable, they must primarily rely on constructs, representing concepts that can be attributed to individuals or groups, and can thus be empirically measured to attain scientific relevance (Markus, 2008). This approach however entails theoretical and methodological problems that are intrinsically related to the subjective and context-bound nature of psychic phenomena, such as the difficulty to reach a single, unified conceptualization and operationalization of the same construct (Rogelberg et al., 2009; Green et al., 2016; Boateng et al., 2018; Park et al., 2022). This issue, specifically evident in positive psychology, does not necessarily represent a limitation, being rather an indicator of the evolution process taking place in this still young research domain; the multiplicity of definitions and measurement tools characterizing several constructs – such as well-being, optimism, resilience and hope, to mention exemplary ones - is a healthy sign of commitment to understand and investigate them by a lively community of scholars approaching the same topic from different perspectives. This exciting exploration phase however implies some disadvantages, such as multiple definitions of the same concept and, as a consequence, development of scales aimed at operationalizing the same concept through the assessment of different facets (Locke, 2012; Podsakoff et al., 2016). In this process, focusing on psychological metrics may lead to disregard the gap between psychological experiences contextualized in real life and their scientific definition and measurement.

This gap specifically characterizes the assumption that mental processes and dimensions unfold along bipolar continua, ranging from low to high values (Danziger, 1990). The epistemological assumption underlying polarization reflects the idea that two theoretical opposites are strictly defined and set off against one another, within a value hierarchy distinguishing between “positive” and “negative” (Smith and Dyer, 1996; Hill and Hall, 2018). This approach is therefore grounded in a specific set of philosophical assumptions about knowledge generation and axiology that influence conceptualizations, confirmation methods, and interpretations (Alexandrova, 2017). Polarization can indeed be useful for articulating categories of elements within highly structured and controlled systems, but it entails the risk of understanding reality as an all-out dichotomized and hierarchical organization of unitary entities. The aggregation of individual data collected through quantitative measurement tools, and their interpretation based on construct related criteria set by researchers, may blur the distinction between a statistically constructed phenomenon and the subjective and context-bound reality that was originally supposed to be investigated (Danziger, 1990).

Moving from the assumption that polarity is an inherent feature of any construct (Pratkanis and Greenwald, 1989; Rossiter, 2011), Tay and Jebb (2018) outlined a two-stage process, labelled continuum specification, aimed to design appropriate psychological measures. The first stage consists in structuring a construct along a continuum, attributing theoretical meaning to the two poles, whereas the second stage allows to define the construct as unipolar, bipolar or combinatorial. Polarized scales are usually in the format of minimum – maximum, totally disagree – totally agree, almost never – almost always. While the upper end corresponds to the complete presence of the construct (totally agree; almost always; maximum intensity), the meaning of the lower end is more problematic, as it may correspond to the absence of the construct (unipolar) or the presence of an opposite construct (bipolar). This controversial aspect often raises disagreement among researchers, leading to the use of both bipolar and unipolar scale formats to measure the same construct (Bagozzi et al., 1999).

The polarization approach is currently challenged by the advancements in “harder” sciences, such as physics and biology (Griffiths, 2012; Kau et al., 2013); its still widespread use in psychology fulfils the aim of representing psychic phenomena as measurable though unobservable entities, a starting point towards a more comprehensive and integrated perspective encompassing qualitative and contextual aspects of the human experience.

### Well-being and mental health as polarized constructs

The challenges of conceptualizing and defining mental health and well-being as psychological constructs clearly emerge in the related literature. In some models they are considered as synonyms with the same meaning; in other ones they are distinguished as constructs differing in broadness, so that one includes the other (Slife et al., 2017; Clifton et al., 2019; Darnell et al., 2019). The World Health Organization (2004) proposed to define mental health as a state of well-being, thus using the two terms as synonyms. Researchers’ conceptual and empirical efforts however led to the identification of specific components of well-being, that were then aggregated into distinct constructs, such as subjective or hedonic well-being (SWB, including positive affect balance and life satisfaction; Diener, 2006) and psychological or eudaimonic well-being (PWB, comprising indicators of positive functioning and development; Ryan and Deci, 2001; Huta and Waterman, 2014; Ryff, 2014). Notably, neither SWB nor PWB represent definitions of mental health, thus contributing to a more refined articulation of an otherwise confused scenario. The empirical evidence emerging from the investigation of SWB and PWB in a variety of populations, and the identification of the construct of social well-being (referring to individuals’ perception of the positive aspects of their societies; Keyes et al., 2002) led to the aggregation of the subjective, psychological, and social well-being indicators into a single construct, considered as adequately representative of the broader concept of mental health (Keyes, 2003, 2007).

Despite these laudable attempts to identify and measure the positive features of psychological functioning, and to distinguish well-being and mental health from the absence of pathological symptoms (Seligman and Csikszentmihalyi, 2000), positive psychology constructs are primarily studied through a context-free approach based on quantification and polarization. Assumed to be purely positive constructs, mental health indicators are described and measured through models juxtaposing their presence versus absence – or maximization versus minimization. To exemplify, in the dual continua model of mental health (Keyes, 2007, 2009), “complete mental health” is the positive condition in which individuals are both “free of mental illness and they are flourishing” (Keyes, 2009, p. 92). Languishing, to the contrary, represents a condition of low or absent mental health. The same polarized juxtaposition is adopted to define and measure single dimensions of positive functioning. For example, measures of optimism are based on its juxtaposition to pessimism (Scheier and Carver, 1992; Peterson et al., 1998). Emotions and affects, components of SWB, are conceptually dichotomized into positive and negative ones (Watson and Tellegen, 1985).

After three decades of investigation of positive psychological constructs, we believe that times are ripe for adopting a more integrated view of mental health, in order to accommodate a variety of epistemologies and empirical findings that are not aligned with the theoretical expectations grounded in a polarized worldview (Uher, 2018b; Holtz, 2020; van Zyl and Salanova, 2022; Veraksa et al., 2022; Wissing, 2022; van Zyl et al., 2023).

### Challenges to the polarization myth

The use of bipolar or dichotomous scales to assess positive mental health facets implies a paradox, as the criteria adopted to distinguish groups of individuals based on their scaled ratings contradict the polarized “all-or-nothing” view. For example, the most frequently used measure of mental health - the Mental Health Continuum-Short Form (MHC-SF, Keyes et al., 2008; Lamers et al., 2011) - includes 14 items referring to psychological facets of emotional or subjective wellbeing (3 items), psychological well-being (6 items) and social well-being (5 items); participants are asked to rate their frequency of occurrence on 6-point scales (0 = never; 5 = everyday). From the measurement perspective, “flourishers” are individuals reporting high frequency ratings (4 or 5) in at least six of the eleven facets of psychological and social well-being (corresponding to eudaimonic functioning) and at least one of the three facets of emotional well-being (the hedonic components). Flourishing thus corresponds to experiencing *some* of the well-being facets on a daily basis, rather than being an all-or-nothing state of complete mental health. Moreover, different flourishers may experience different well-being facets with daily frequency, giving rise to different flourishing patterns. On the other hand, people with moderate mental health can still experience up to five facets of well-being daily. This paradox characterizes most multidimensional measures of well-being, such as the Psychological Well Being Scales (Ryff, 1989; Ryff and Keyes, 1995), the Meaning in Life Questionnaire (Steger et al., 2006), the Basic Psychological Needs Scale (Gagné, 2003), and the Positive Affect Negative Affect Schedule (Watson et al., 1988). In addition, for most of these measures no cut-off values are provided to categorize different well-being conditions along the scales.

Interestingly, studies conducted across cultures and life stages allowed for detecting variegated configuration patterns of mental health components, in terms of both blend and intensity levels, across individuals and groups as well as within the same person over time. These varying patterns emerged for the Psychological Well Being Scales (Ryff et al., 2002; Pancheva et al., 2021), the MHC-SF (Westerhof and Keyes, 2010), the Basic Psychological Needs Scale (Ryan and Deci, 2000; Ginevra et al., 2015), and the Satisfaction with Life Scale (Diener et al., 2013; Joshanloo and Jovanović, 2018; Krys et al., 2021; Schutte et al., 2021). These findings show the importance of looking beyond the quantitative interpretation of values – be they referring to single items, dimensions, or whole scales - in order to identify qualitative configuration patterns that may characterize specific individuals in specific societies, communities, and/or life stages, allowing for a more comprehensive and contextualized understanding of the underlying constructs (Goertz and Mahoney, 2012; Carlquist et al., 2018).

At the population level, the difficulties entailed in an “all-or-nothing” view of mental health, and in the interpretation of related results, emerged in the European Social Survey data. Huppert and So (2013) proposed a model of flourishing that includes a set of positive dimensions conceptually defined as the “mirror opposite” of the major affective disorder symptoms described in the Diagnostic and Statistics Manual (DSM-IV). The mean scores of each dimension, measured on a bipolar scale, showed a specific rank distribution pattern across countries. Each country was characterized by a peculiar mixture of high, intermediate, and low ratings for different dimensions, rather than showing a global polarization towards one single end of the ranking. For example, France ranked first for engagement, and last for self-esteem; Spain ranked first for self-esteem, and last for vitality. These country-specific, multifaceted patterns of mental health can only be interpreted in terms of qualitative configuration patterns, emerging from the complex interplay of psychological dimensions with demographic, socioeconomic and linguistic aspects, as well as national inequality levels, cultural values, and social policies.

Consistent with this evidence, across disciplines well-being maximization is increasingly considered as a utopian and/or a culturally biased goal (Bacon, 2005; Grant and Schwartz, 2011; Kjell, 2011; Wong, 2012; Lelkes, 2013), just like the definition of health as a complete state of bio-psycho-social well-being (World Health Organization, 1946). Most individuals across countries report intermediate levels of positive mental health, partly determined by contextual and demographic factors. For example, in most studies conducted through the MHC-SF around 50% of the participants turn to be “moderately healthy” – neither flourishing nor languishing (Keyes, 2007; Keyes et al., 2008; Petrillo et al., 2015; Bassi et al., 2021). Similarly, over 60% of US participants examined through SWB and PWB measures (Ryff et al., 2002; Pancheva et al., 2021), reported either overall moderate levels of both, or different combinations of high, moderate and low levels of SWB and PWB. Only in a minority of cases SWB and PWB showed polarized combinations (very high vs. very low; both very high; both very low). These findings suggest the heuristic potential of investigating differences and similarities in the configuration patterns of well-being dimensions characterizing individuals beyond their position along a quantitative continuum of mental health or optimal functioning. Such an analysis can shed light on the contribution of single components of well-being to different configuration patterns, on the joint recurrence of some components across patterns, taking into account participants’ personal and contextual features.

Finally, the empirical literature shows that maximization of any well-being dimension may be harmful. The search for increasingly higher pleasure and extrinsic rewards generates the hedonic treadmill (Diener, 2000). An excessive dedication to activities associated with flow experience (Csikszentmihalyi, 1975) may lead to physical impairments (as detected among musicians; Guptill, 2012), or neglect of other life domains (Delle Fave et al., 2011b). Overfocus on a single interest may lead to obsessive and thus maladaptive passion (Vallerand et al., 2003). Consequences of excess related to positive psychological dimensions are however still underexplored.

## The contexts and dynamics of mental health

This section is focused on some neglected features of mental health as a multidimensional and intrinsically dynamic phenomenon, that undergoes changes with time and through the ceaseless interplay between individuals and their environment. Emphasis will be put on both the individual and contextual factors contributing to shape mental health. As concerns the dynamic nature of mental health, specific attention will be devoted to balance, a core feature of living beings and, consequently, of human psychological processes. Far from being a static state like homeostasis, dynamic balance plays a substantial role in mental health; its investigation may allow to overcome the polarization perspective. To this purpose, the identification of specific configuration patterns of well-being components within multidimensional constructs may represent a useful starting point.

### The multidimensional nature of mental health: individual and contextual features

A variety of personal and environmental features can contribute to the individual ratings of positive psychological dimensions, challenging the approach of maximization versus minimization, presence versus absence of mental health.

As concerns individual features, high correlations were recurrently detected between mental health ratings and personality traits (Diener and Lucas, 1999; Ryff et al., 2002; Keyes, 2007; Steel et al., 2008; Weiss et al., 2008; Lucas and Diener, 2009). To exemplify, extraversion is more frequently associated with flourishing compared to introversion; neuroticism is often associated with lower subjective well-being levels. These correlations led several scientists to consider positive facets as trait-like or even genetically determined dimensions (Gallagher et al., 2009; Cummins et al., 2014; Keyes et al., 2015).

Over and above personality, factors directly or indirectly related to individual developmental processes (such as physical characteristics, age and life stages) and environmental features (such as socio-economic status, life circumstances, and culture) may contribute to variations in positive psychological dimensions. Longitudinal findings showed that major negative life events may undermine positive affect (Hentschel et al., 2017). A study using Gallup World Poll data (Oishi and Diener, 2013) highlighted that citizens of poorer countries perceive lower satisfaction with life, but higher meaning in life than citizens of richer countries. People experiencing chronic diseases report dissatisfaction and low positive affect, but high levels of meaning, resilience, and post-traumatic growth (Andrykowski et al., 2008; Lechner et al., 2009; Bassi et al., 2014). Age related biological and social conditions are associated with different combinations of PWB facets’ ratings (Ryff, 2014); for example, in both Japan and the US older participants report lower levels of purpose in life but higher levels of autonomy, compared with youth (Karasawa et al., 2011). Moreover, across studies older participants usually report intermediate ratings of both emotional and cognitive dimensions of well-being, a tendency attributed to the acquisition of a more balanced view of reality, also called wisdom by some scholars (Ryff et al., 2002; Mogilner et al., 2011; Delle Fave et al., 2013a,b).

At the broader societal level, cultural assumptions about reality, personhood and science may give prominence to different components of mental health, thus influencing its evaluation (Thompson et al., 2020). Cultural variations in mental health definitions specifically call into question the bipolar approach and the view of individuals as the primary units of analysis, typical of the Western context in which most psychological theories and models were developed (Henrich et al., 2010). For example, in South and East Asian contexts polarization is deemed as inadequate to represent both objective reality and psychological phenomena. Contrasting qualities, such as positive and negative or good and bad, are conceptualized as complementary and interacting characteristics of reality; consequently, the “exclusively positive” is not endowed with an intrinsically positive value and meaning. In the philosophical traditions of Hinduism, Taoism and Buddhism, the universe is represented as a balanced system, in which different complementary elements harmoniously coexist and dynamically interact with each other (Salagame, 2013). At the psychological level, states of moderation and evenness - the “middle beyond extremes” - are praised over intense emotions, extreme experiences, and peak performances (Dharmachakra Translation Committee, 2006). Such an emphasis on a blended coexistence of more entities, rather than a polarized prominence of single units, emerges from the findings of psychological studies conducted in these regions (Leung et al., 2002; Huang, 2016). These ontological perspectives, culturally constructed and historically transmitted, shape the understanding and conceptualization of mental phenomena (Markus and Kitayama, 1991; Igbokwe and Ndom, 2008; Ohajunwa and Mji, 2018), leading to the development of constructs such as “interdependent happiness” (Hitokoto and Uchida, 2015), or to the investigation of culturally specific concepts like “Ubuntu,” indicating that a person’s humanity is inextricably linked to the humanity of another (Nyamnjoh, 2019). At the social level, these views are reflected in the collectivistic organization of most African and Asian communities (Triandis, 1974; Hofstede, 1980; Igbokwe and Ndom, 2008; Lim, 2009; Uchida and Ogihara, 2012), characterized by the primacy of relational aspects over pursuit of individual goals and desires. The positive features of individuals, community, nature, and spiritual forces are conceived as reciprocally balanced and harmoniously intertwined (Igbokwe and Ndom, 2008; Lim, 2009; Huang, 2016; Nwoye, 2017).

Overall, these findings point to the need for more accurately contextualizing positive dimensions of mental health (Di Martino et al., 2017). Moreover, they suggest that the interplay between personal and environmental variables in shaping individuals’ mental health is multidimensional and often bidirectional (Neyer and Lehnart, 2007; Kern and Friedman, 2011; Friedman et al., 2013). Human beings and their environments are complex and dynamic entities undergoing changes over time. Individuals may progressively achieve better or worse levels of adjustment to external conditions, by adapting their behavior to the environment either through accommodation and secondary control, or through agency and primary control. The environment undergoes changes as well, thus requiring individuals to gradually modify their adjustment strategies, or to develop new ones in the face of sudden and disrupting events (Terry and Hynes, 1998; Joseph and Linley, 2005). It is thus not surprising that individuals’ evaluations of mental health components, as well as their configuration patterns, may vary along a developmental trajectory, shaped by their everchanging interplay with the environment.

### Dynamic balance as a core feature of living systems

The development of a positive mental health conceptualization endorsing change and adaptation processes is consistent with the intrinsically dynamic nature of reality. Studies in epigenetics (Lachmann and Jablonka, 1996; Zhang and Meaney, 2010) highlighted that the adaptive interaction of the organism with the environment may produce changes in gene expression and biological structures, which can be transmitted across generations to enhance behavioral plasticity and flexibility (Masterpasqua, 2009). These studies challenge the assumption of homeostasis (or static balance) as a fixed and time independent set-point, around which biological processes revolve, and to which organisms return whenever possible, after deviations due to environmental or internal pressures.

Consistent with this dynamic view is the concept of *homeorhesis*, defined as the tendency to follow a developmental trajectory that may lead to changes in a system’s setpoint (Waddington, 1957). The related term *chreod* represents the trajectory (rather than the set-point) to which a system tends to return. The developmental chreod characterizing individuals is shaped by the unique and ceaseless interaction between their stable or quasi-stable traits, the environment(s) to which they are exposed during life, and random events that may take place at the individual and environmental levels (Lewontin, 2000; Fusco and Minelli, 2010). Homeorhesis is a necessary feature of living systems. It can be defined as “piecewise homeostasis,” in that it allows the system to rejoin the adaptive trajectory after deviations. This trajectory may however change direction with time, leading to changes in the set-point to which the system tends to return (Mamontov, 2007). These biological mechanisms can be logically transposed to the psychological level. Personality, cognitive and emotional processes, skill development, goal setting and environmental factors interact together in building an individual pathway of development open to change and unpredictability, and characterized by time-varying, nonperiodic states rather than stability or maximization (Glass, 2001).

### Beyond polarization: dynamic balance in psychological processes

Learning is the core mechanism through which living beings dynamically adapt to environmental changes. It unfolds into a variety of patterns, from conditioning to insight and creative thinking. The adjustment process guided by learning is dynamic in nature, and it may imply deviations from time independent homeostasis, as well as changes in the system’s organization and functioning.

Several psychological models more or less implicitly assume dynamic balance as the core of learning. A typical example is optimal discrepancy (Piaget, 1936), that orients infants’ attention towards stimuli which are neither too similar, nor too different from the familiar ones, giving rise to the learning trajectories of assimilation or accommodation, the latter promoting changes in the categorization process. Moving to well-being studies, dynamic balance is an intrinsic feature of flow experience (Csikszentmihalyi, 1975). The balance between high levels of perceived challenges and adequate personal skills in facing them is a key prerequisite for flow to occur during an activity. This balance is intrinsically dynamic, as skills tend to increase through practice, leading individuals to search for more complex challenges to preserve flow experience in the same activity over time. This virtuous cycle orients psychological selection (Csikszentmihalyi and Massimini, 1985), namely the preferential cultivation of a specific set of activities along the person’s developmental trajectory. Psychological selection is however characterized by homeorhesis, as life events may modify individual’s skills, motivations, and/or environmental features, both quantitatively and qualitatively. Based on a variety of configurations of contextual and personal conditions, individuals can discover new or previously ignored activities as opportunities for flow. They can thus decide to engage in the selective cultivation of these activities, with consequent changes in the typology of physical, mental an social skills preferentially developed and refined over time (Massimini and Delle Fave, 2000; Delle Fave and Massimini, 2005). These examples – among many others easily identifiable in the psychological literature - underscore the need for considering the intrinsically dynamic nature of human beings when attempting to measure any psychological dimension or process, including mental health.

## Towards a new definition of mental health

This section summarizes international evidence suggesting that balance and harmony are core qualities of mental health, shared across cultures. Based on these findings, a new definition of mental health is proposed, with harmonious balance as its core constituent.

### Balance and harmony as core qualities of mental health

In psychological research, balance and harmony were found to be semantically related to tranquility, peace, concord, agreement and cooperation (Kjell et al., 2013). In the Asian context of Taoism and Hinduism, they represent the pattern along which the universe naturally unfolds and differentiates from the original condition (Li, 2008; Bhawuk, 2011). At the psychological level, the Hindu view praises *anasakti*, the ability to preserve non-attachment and equipoise under both favorable and unfavorable circumstances (Gotise and Upadhyay, 2017). In the Confucian view, the practice of different virtues is not valuable by itself, unless it leads to the harmonious combination of these virtues, so that “harmony results in happiness” (Liu, 2003, p. 71).

Interestingly, also in the Western tradition mental health was described as balance and harmony rather than maximization of the positive, starting from the ancient Greek philosophers (Michalos and Robinson, 2012; Delle Fave, 2021). Epicure focused on ataraxia as freedom from worries or anxiety, through the ability to maintain balance and equipoise under good and bad life circumstances (in line with the Hindu concept of *anasakti*). Heraclitus described harmony as a condition of positive balance emerging from the interaction between opposing tensions. He also highlighted the intrinsically impermanent and ever-changing nature of reality, synthesized in the sentence “παντα ῥεῖ”: everything flows. Stoics underscored the “detached acceptance” of life’s joys and hurdles, connecting it to the ideal of evenness of judgment. Pythagoras put harmony at the core of his philosophy of numbers. Plato defined mental health as *sophrosyne*, a term composed by σωσ = whole, sound, saved; and φρην = mind, representing moderation, self-control, and self-awareness, contrasted with ύβρις = pride, overconfidence. Aristotle (1985) used this same term in the *Nichomachean Ethics* (*ca.* 340 B.C.E.), referring to the virtue of moderation or temperance. Moreover, his overall conceptualization of virtues is rooted in the concept of mean between extremes (Grant and Schwartz, 2011). The convergence with the Buddhist and Confucian views is remarkable (Sim, 2004).

Despite this transcultural philosophical legacy, and despite the recurrent identification of dynamic balance as a core psychological mechanism, positive psychology researchers only recently addressed harmony and balance as dimensions or qualities of mental health. Mixed-method studies based on a bottom-up approach, and involving adults from different countries (Delle Fave et al., 2011a, 2016) showed that, when asked to define happiness in their own words, participants primarily referred to inner harmony and balance at the psychological level, and to interpersonal relationships at the contextual level. Inner harmony as a broad category comprised more specific dimensions such as contentment, serenity, equipoise, and self-acceptance. Relationships with family, friends and significant others were described as intrinsically valuable contexts characterized by sharing and reciprocity. Similar findings were obtained from people with chronic diseases and their caregivers (Delle Fave et al., 2017).

Psychological balance and flexibility were subsequently operationalized and quantitatively measured through the Harmony in Life Scale (Kjell et al., 2016), aimed at complementing the cognitive components of subjective well-being. This formulation of intrapersonal harmony was then extended to capture the social component of subjective well-being as the balance between the individual and the surrounding context (Nima et al., 2020).

The concept of balance was also elaborated as a dimension of the self-environment relationship. Dambrun (2017) conceptualized two different orientations towards this relationship, representing separate constructs rather than opposite poles of a continuum, and showing different associations with well-being. Selflessness, which implies harmonious adjustment with other people and the environment, is related to the eudaimonic construct of authentic-durable happiness (Dambrun and Ricard, 2011). Self-centeredness, which implies separation between self and the rest of the world, is associated with the emotion-based construct of fluctuating happiness and with higher anxiety. The focus on others versus self also characterizes the quiet ego (Wayment et al., 2015; Wayment and Bauer, 2018), a representation of self that derives from the balance between self-interest and concerns for others.

With a focus on balance, Wong (2011) proposed a model of good life based on the dual-systems model of avoidance and approach motivation, which integrates the complexities of the negatives and positives in such a way that adaptive positive outcomes can be obtained in all kinds of situations, and a balance is found between individualist and collectivist cultural orientations. Wong and Bowers (2018) explicitly linked this idea to the concept of mature happiness and harmony, proposing a related measurement scale. Sirgy (2019) formulated a hierarchical model of mental health described as a positive balance, in which there is a preponderance of desirable versus undesirable elements at six levels - physiological, emotional, cognitive, meta-cognitive, developmental and social- ecological. In a narrative review, Lomas (2021) categorized harmony and balance in terms of affect, cognition, behavior, and self-other relations.

All these definitions of harmony and balance share some core features. They refer to a positive psychological condition of low arousal and equilibrium, that can be altered by external or internal pressures leading to imbalance, but also restored along the developmental trajectory. Moreover, defining harmony and balance as core dimensions of positive mental health intrinsically endorses change, as it implies a dynamic process of adjustment, learning, and integration over time, through the progressive acquisition of information, competences, and experiences. It also encompasses adjustment to life circumstances that force individuals to revise their goals and meanings. In addition, all these models endorse the interconnectedness between individuals and their social, natural and global environment (Kjell et al., 2016; Delle Fave et al., 2022). Overall, they highlight the specific contribution of harmony and balance to a more comprehensive and holistic view of mental health. This evidence led to the inclusion of harmony/balance among the dimensions assessed in the most recent data collection wave of the World Happiness Report (Lomas et al., 2022).

### Harmonious balance as mental health

The evidence of harmony and balance as positive psychological dimensions is aligned with the most recent formulations of mental health developed in the domain of psychiatry. Against the maximization view of mental health as a complete state of well-being, Bhugra et al. (2013) proposed to define it as “a state of equipoise where individuals are at peace with themselves, are able to function effectively socially and are able to look after their own basic needs as well as higher function needs. Positive functionality means managing change, relationships and emotions in a constructive manner” (p.3). In the same vein, Galderisi et al. (2015) suggested to define mental health as “a dynamic state of internal equilibrium which enables individuals to use their abilities in harmony with universal values of society. Basic cognitive and social skills; ability to recognize, express and modulate one’s own emotions, as well as empathize with others; flexibility and ability to cope with adverse life events and function in social roles; and harmonious relationship between body and mind represent important components of mental health which contribute, to varying degrees, to the state of internal equilibrium” (p.231–232).

The conceptual and empirical evidence discussed in the previous sections suggests that a dynamic condition of harmonious balance can describe mental health better than maximization of either single dimensions or combinations of variables. It is also more consistent with the real-life dynamics of individual/environment interactions, that contribute to shaping individuals’ life trajectories, goal setting and pursuit, and meaning making (Baumeister et al., 2011; Bassi and Delle Fave, 2016).

At the cross-disciplinary level, the view of mental health as dynamic balance is aligned to the evaluation of physical health as optimal balance among multiple biological parameters’ values (not too low, not too high), and as a dynamic condition requiring continuous adjustments based on gender, life stages, disease conditions, and environmental pressures. At the cross-cultural level, this perspective brings together different views of psychophysical health, including those characterizing the Chinese and Indian traditional medicines, in which the healthy body–mind system is described as an integrated and dynamically balanced entity (Meulenbeld, 2000).

We therefore propose to define mental health as “a condition in which individuals perceive a harmonious and dynamic integration among the different parts of themselves; contentment and inner peace; a balance in concerns for self and others; a dynamic presence of meaning; a balanced interplay between personal desires/goals/responsibilities and environmental challenges and opportunities in relevant life domains.”

This definition encompasses all the key dimensions of well-being identified by the literature, focusing on their balance rather than maximization, and on their harmonious interplay within an integrated perspective. More specifically, the perceived self-integration endorses self-acceptance and good relationship with oneself, dimensions included in eudaimonic models of well-being (Ryff, 1989; Wong, 2011). In line with Dambrun et al. (2012), Bhugra et al. (2013), and Wong and Bowers (2018), contentment and inner peace refer to equipoise, rather than to maximization of positive emotions and avoidance or minimization of negative ones. The balance in concerns for self and others endorses the constructs of authentic-durable happiness (Dambrun, 2017) and quiet ego (Wayment and Bauer, 2018). The inclusion of presence of meaning as a dynamic dimension underscores its interplay with the search for new meanings, and therefore its intrinsic potential for constructive change and evolution (Steger et al., 2006). Finally, the dimension of balanced interplay between personal desires/goals/responsibilities and environmental challenges and opportunities in relevant life domains refers to the adaptive and balanced interaction of individuals with their life context, in terms of coping with external demands, identifying and pursuing goals, and adequately invest and distribute psychophysical resources across the relevant life domains (Baumeister et al., 2011; Delle Fave et al., 2011b; Galderisi et al., 2015; Kjell et al., 2016; Sirgy, 2019). In addition, this definition is substantially aligned with the views of well-being and mental health endorsed by the philosophical traditions of different cultures, as briefly outlined in this section.

The proposed definition of mental health relies upon three major principles: it endorses both qualitative and quantitative changes, as it implies a dynamic process of adjustment and integration over time and through the progressive acquisition of diverse skills, competences and life experiences; it takes into account the natural occurrence of negative events and environmental circumstances that force individuals to revise their plan and goals, integrating positives and negatives in the real life context; and it holds up across life domains, such as work, family, health, community/society, and spirituality/transcendence.

### Implications for research and intervention

The potential of focusing on harmonious balance as mental health in the context of well-being studies and positive psychology is threefold. At the theoretical level, it can orient the attention of researchers towards qualitative configuration patterns of well-being dimensions. Exploring the ways in which different people describe the interplay between different components of well-being and positive mental health (beyond focusing on their higher or lower values, and on the general distinction between hedonic and eudaimonic dimensions) may shed light on their varying configuration patterns across individuals and groups, as well as on the dynamic process of mental health building and unfolding across environmental and person-related conditions. This approach could also provide information on the different strategies adopted by people across contexts and life stages in preserving and promoting a constructive relationship with themselves and their environments. It could allow for identifying similarities and differences in these adaptation patterns across groups, based on the qualitative interplay between different dimensions, thus integrating quantitative comparisons.

At the methodological measurement level, the exploration of configuration patterns of well-being dimensions can fruitfully benefit from the use of advanced statistical clustering approaches, such as Latent Class/Profile Analysis (LPA/LCA), which allow for detecting profiles of individuals sharing similar patterns of well-being dimensions (Spurk et al., 2020). For example, it will be possible to identify different profiles of flourishing individuals, based on the daily recurrence of different sets of the mental health components investigated by the MHC-SF; it will be possible to shed light on the understudied though vast population of individuals reporting moderate levels of mental health; cultural, social and personal predictors could be identified for each profile. In so doing, it will be possible to focus on the interplay between different components of well-being within each single mental health category of individuals, identified through bipolar scales, shedding light on the qualitative contribution of specific components to each profile, thus defined as a more or less balanced set of well-being components. Another technique that could shed light on the interplay between different well-being components within the same mental health category is the self-organized map (SOM) neural algorithm (Kohonen, 2001), that allows to inspect patterns underlying complex multidimensional phenomena, and that was recently used to investigate the distribution of hedonic and eudaimonic dimensions of well-being within the MIDUS database (Pancheva et al., 2021).

As concerns the investigation of mental health as a dynamic process, ambulatory assessment can provide qualitative and quantitative information, allowing for the integration of idiographic and nomothetic principles of measurement (Wright and Zimmerman, 2019). This approach is centered on the collection of information about the subjective experiences of individuals, as they unfold during daily activities and contexts (enabling ecological validity), in real time (minimizing retrospective biases) and on repeated occasions (longitudinal approach). Ambulatory assessment covers a range of methods, such as experience sampling method, ecological momentary assessment, real-time data capture and e-diary. This approach has been fruitfully employed for the study of experience fluctuations across daily activities and contexts, leading to the identification of experiential states – such as flow – represented by specific configuration patterns of experience components, interacting with each other in a more or less harmonious and balanced way (Hektner et al., 2007; Delle Fave and Bassi, 2022). Multiple assessments across time and within individuals enable researchers to investigate how psychological processes and behaviors evolve, vary, and relate to one another over time (Ryan et al., 2018). In order to analyze this kind of intensive longitudinal data sets, however, advanced statistical techniques are required, such as structural equation modelling, multilevel modeling, sequential analysis, and dynamic systems analysis and machine learning, which allow to handle large amounts of data and provide powerful insights into dynamic processes (Wright and Hopwood, 2016).

At the intervention level, an approach focused on the dynamic interplay of different components of well-being within specific, and more or less balanced configuration patterns, may lead to the personalization of well-being promotion – a perspective that is, most likely, endorsed and practically adopted by professionals and practitioners in their daily work with patients, but that would substantially benefit from a scientific support by researchers.

Apart from the integration of conceptual, methodological and measurement levels (including the use of sophisticated statistical analytical techniques) in psychological research and intervention, epistemological approaches and methods from transdisciplinary sciences also need to be explored in future, as indicated by Uher (2018b), taking into account the basic principles of data generation traceability as well as numerical traceability which is pertinent across sciences (Uher, 2018a). Pursuing this goal however requires a more comprehensive and less discipline-bound view of well-being, which can capture the universal tendency of living systems towards inner coherence and balance – on the one side – and towards dynamic growth and interconnectedness, on the other side. Researchers should be ready to acknowledge the boundaries of their field-specific knowledge and engage in cooperative efforts, towards the building of a truly integrated view of reality (Delle Fave, 2016).

## Concluding remarks

By discussing the limitations of the polarization perspective and related methods in the investigation of psychological phenomena, including mental health, we have adopted the philosophical critical realist perspective and view of reality as a complex, interconnected system (Nicolescu, 2014a,b), dynamically changing and evolving over time (Gadamer, *cf.* Malpas, 2018). The main assumption of this view is the existence of an independent reality that can be known only to a certain extent; this knowledge is influenced by context, human imperfections, and human fallibility. Therefore, we humbly accept that our view on harmonious balance as a core quality of mental health is based on available models and theories, empirical findings, and our current ontological and epistemological assumptions. A challenge for future research in this field is to investigate harmonious balance and related processes from various epistemological approaches relying on different methodologies, and to benefit from the complementarity of transdisciplinary approaches, as also argued by Uher (2018b). The way towards this kind of integration is however still to be clearly traced, as it requires the joint effort of researchers from different disciplinary fields, joining forces to build a more unitary and shared view of human psychic, behavioral and social processes. We finally acknowledge that knowledge itself – as well as related world view assumptions - undergoes changes over time in any science, including psychology.

## Data availability statement

The original contributions presented in the study are included in the article/supplementary material, further inquiries can be directed to the corresponding author.

## Author contributions

All authors listed have made a substantial, direct, and intellectual contribution to the work, and approved it for publication.

## Conflict of interest

The authors declare that the research was conducted in the absence of any commercial or financial relationships that could be construed as a potential conflict of interest.

## Publisher’s note

All claims expressed in this article are solely those of the authors and do not necessarily represent those of their affiliated organizations, or those of the publisher, the editors and the reviewers. Any product that may be evaluated in this article, or claim that may be made by its manufacturer, is not guaranteed or endorsed by the publisher.
